# Postbiotics enhance the efficacy of derivative compound mouthwash against clinical *Helicobacter pylori* strains

**DOI:** 10.3389/fcimb.2025.1629106

**Published:** 2025-07-23

**Authors:** Rawee Teanpaisan, Nuntiya Pahumunto

**Affiliations:** ^1^ Medical Science Research and Innovation Institute, Prince of Songkla University, Hat Yai, Thailand; ^2^ Research Center of Excellence for Oral Health, Faculty of Dentistry, Prince of Songkla University, Hat Yai, Thailand; ^3^ Department of Oral Diagnostic Sciences, Faculty of Dentistry, Prince of Songkla University, Hat Yai, Thailand

**Keywords:** postbiotics, mouthwash, derivative compound, glycerol monolaurate, *Helicobacter pylori*

## Abstract

**Background:**

A previous study indicated that poly L-lysine-glycerol monolaurate mouthwash reduced the virulence of *Helicobacter pylori*; however, these compounds are derivatives. Thus, this study aimed to compare the effects of postbiotics, postbiotic-glycerol monolaurate, and poly L-lysine-glycerol monolaurate mouthwashes against clinical *H. pylori* strains.

**Methods:**

Postbiotics, *Lacticaseibacillus paracasei* SD1, *L. rhamnosus* SD4, and *L. rhamnosus* SD11 were examined for anti-bacterial activity and synergistic effects. Subsequently, mouthwashes containing postbiotics, postbiotic-glycerol monolaurate, and poly L-Lysine-glycerol monolaurate were prepared and evaluated for their ability to reduce *H. pylori* adhesion to host cells, suppress inflammation induced by *H. pylori*, eradicate biofilm, decrease *cag*A expression, and assess epithelial cell viability. The stability of the mouthwashes was evaluated every 4 weeks up to 24 weeks for their efficacy against *H. pylori* growth, biofilm eradication, and epithelial cell viability.

**Results:**

The postbiotics, *L. paracasei* SD1 and *L. rhamnosus* SD11, demonstrated significant anti-*H. pylori* activity, with synergistic effects observed in combinations with derivative compounds. Postbiotic-glycerol monolaurate mouthwashes exhibited higher efficacy in reducing *H. pylori* adhesion to host cells (42.64-43.83%), suppressing pro-inflammatory cytokines, eradicating biofilm (82.62% at 24 h), and reducing *cag*A expression (112.60 fold) compared to others. Such mouthwashes also displayed low cytotoxicity (< 30% for 15 min) to all cells tested. The stability was observed up to 24 weeks.

**Conclusion:**

This *in vitro* study demonstrated that postbiotic-glycerol monolaurate mouthwash revealed the highest efficacy against *H. pylori* with low cytotoxicity to host cells. The stability lasted for 24 weeks.

## 1 Introduction

*Helicobacter pylori* is found in more than 50% of the global population and has been linked with gastritis and gastric cancer (Zamani et al., 2018). In our previous study, the prevalence of *H. pylori* in the oral cavity of participants ranged from 83.92% to 86.73%, which was detected by a polymerase chain reaction (PCR). Among patients with gastritis, the prevalence was observed to be between 93.33% and 100.00%, while in gastric cancer patients, it ranged from 80.00% to 86.67% as determined by a PCR (Wongsuwanlert et al., 2024a). The results suggested that the oral cavity may serve as a reservoir for *H. pylori* or a means of transmission to other sites. Furthermore, studies found that oral *H. pylori* infections are associated with a high gastritis recurrence rate (13.20%-18.40%) (Yee, 2017; Sheu et al., 2007). Thus, combining oral and gastrointestinal therapies enhances the effective treatment of *H. pylori* infection (Wang et al., 2014).

Standard treatment for *H. pylori* infection is triple therapy, which combines two antibiotics (clarithromycin and either amoxicillin or metronidazole) with a proton pump inhibitor (Chey and Wong, 2007). *H. pylori* strains have demonstrated significant antibiotic resistance, particularly to metronidazole, while bismuth compounds exhibit high toxicity to host cells, which complicates treatment further (Wongsuwanlert et al., 2024a). Additionally, treatment of *H. pylori* infection focuses on removing *H. pylori* from the stomach, but there is limited data on eliminating *H. pylori* in the mouth. A previous study by Wang et al. (2014) demonstrated that a mouthwash containing poly L-lysine and glycerol monolaurate reduced oral *H. pylori* levels in volunteers. Specifically, the mouthwash decreased *cag*A mRNA expression, reduced bacterial adhesion, inhibited *H. pylori* growth, and lowered interleukin-8 expression (Wongsuwanlert et al., 2024b). Glycerol monolaurate is a derivative compound recognized for its beneficial effects for food preservation and oral hygiene products (Wang et al., 2014; Wongsuwanlert et al., 2024b). In contrast, poly L-lysine has several limitations, including its high cost, significant toxicity to epithelial cells, reliance on chemical synthesis, lack of biodegradability under all environmental conditions, and complex polymerization processes (Wang et al., 2014; Chen et al., 2021). There is significant interest in researching the effects of organic substances as alternatives to replace derivative compounds to develop safer and more effective therapeutic agents targeting *H. pylori*. Meta-analyses of systematic reviews have shown that probiotic supplementation was significantly associated with increased *H. pylori* eradication rates by 10% compared to the control group and showed a lower risk of overall side effects by 46% compared to standard therapy (Yang et al., 2024; McFarland et al., 2016). In addition, consuming probiotics before or after triple treatment can enhance the therapeutic effects for individuals infected with *H. pylori* (Wang et al., 2023). Postbiotics have recently gained considerable popularity. They are preparations of inanimate microorganisms or their components including cell-free supernatants, cell walls, and metabolites (Salminen et al., 2021). Postbiotics from *Lacticaseibacillus paracasei* SD1 and *L. rhamnosus* SD11 have been shown to inhibit pathogen growth and reduce pro-inflammatory cytokines with effects comparable to those of live probiotics *in vitro* and *in vivo* (Wanitsuwan et al., 2024; Pahumunto and Teanpaisan, 2023). Postbiotics from *L. paracasei* SD1, *L. rhamnosus* SD4, and *L. rhamnosus* SD11 have been shown to inhibit *H. pylori* adhesion to oral epithelial cells (Juntarachot et al., 2023). The inactivated *Limosilactobacillus reuteri* DSM17648 improves *H. pylori* eradication in patients with functional dyspepsia and decreases digestive adverse effects (Ivashkin et al., 2024). However, Yang et al. (2021) found that non-viable *L. reuteri* DSM17648 with triple therapy did not improve eradication rates; however, it promoted a beneficial microbial profile and a decline in gastrointestinal symptoms. Thus, it remains necessary to identify the most effective postbiotics for *H. pylori* treatment. This study aimed to examine the anti-bacterial ability of selected postbiotic strains against oral *Helicobacter pylori* strains. Subsequently, this study compared the effects of postbiotics-, postbiotic-glycerol monolaurate-, and poly L-Lysine-glycerol monolaurate- mouthwashes against oral *H. pylori* strains.

## 2 Materials and methods

### 2.1 Bacterial preparation

#### 2.1.1 Probiotic cultivation

The selected probiotic strains were *Lacticaseibacillus paracasei* SD1, *L. rhamnosus* SD4, and *L. rhamnosus* SD11, which were isolated from the oral cavity of individuals with a caries-free status. These strains were cultured on de Man, Rogosa, and Sharpe (MRS) agar (Difco™, Sparks, MD, USA) at 37°C for 24 h under anaerobic conditions (10% CO_2_, 10% H_2_, 80% N_2_). These optimal conditions were observed in the log phase of growth for the probiotic strains (Juntarachot et al., 2023; Wannun et al., 2014). Probiotic cells were collected using centrifugation at 10,000 rpm for 10 min at 20°C, and the cells were adjusted to 8 log CFU/mL before testing.

#### 2.1.2 Postbiotic preparation

Probiotic strains (8 log CFU/mL) were cultured in de Man, Rogosa, and Sharpe (MRS) broth (Difco™, Sparks, MD, USA) at 37°C for 24 h under anaerobic conditions (10% CO_2_, 10% H_2_, 80% N_2_) based on our previous studies that tested incubation times at 12, 24, 48, and 72 h (Thananimit et al., 2022). A 24 h incubation was identified as the optimal condition for promoting the production of bioactive compounds including anti-oxidant, short-chain fatty acids, and antimicrobial proteins (Chooruk et al., 2017; Thananimit et al., 2022; Wannun et al., 2014). The postbiotics were collected by centrifugation at 10,000 rpm for 10 min at 4°C. They were then filtered using a 0.45 µm syringe filter (Sartorius stedium, Germany) and lyophilized using a freeze dryer. The postbiotic powder was kept at -80°C until usage.

#### 2.1.3 *Helicobacter pylori* cultivation

The study was approved by the Ethical Committee of the Faculty of Dentistry, Prince of Songkla University (EC6410-066). *H. pylori* ATCC43504 and nineteen clinical strains of *H. pylori* isolated from the oral cavity (saliva and plaque) were used throughout the study. Clinical strains were confirmed using a polymerase chain reaction with specific primers (Wongsuwanlert et al., 2024a). *H. pylori* strains were cultured on brain heart infusion (BHI) agar (Difco™, Sparks, MD, USA) supplemented with 5% blood at 37°C for 48 h in anaerobic conditions (Juntarachot et al., 2023; Perez-Perez et al., 2016). *H. pylori* strains (3–5 colonies) were picked up to re-streak a full plate on BHI agar, and *H. pylori* cells were collected for culture in BHI broth (Difco™, Sparks, MD, USA) for 48 h at 37°C in anaerobic conditions (Juntarachot et al., 2023; Wongsuwanlert et al., 2024b). Then *H. pylori* cells were collected using centrifugation at 10,000 rpm for 5 min and adjusted to 8 log CFU/mL to be used in all experiments.

### 2.2 Screening of anti-*H. pylori* activity of probiotics and postbiotics using agar well diffusion and agar overlay methods

#### 2.2.1 Agar well diffusion method

An *H. pylori* strain at 8 log CFU/mL was added to 20 mL of melted BHI agar and poured on a plate with metal cups. After solidifying, the metal cup was removed and postbiotic solutions (postbiotic powder 1 mg in 1 mL distilled water) or derivative compounds (1 mg of poly L-Lysine or glycerol monolaurate in 1 mL distilled water) (Sigma, St. Louis, MO, USA) were added to each well. The plate was incubated overnight at 37°C in anaerobic conditions. The inhibition zone was measured and reported as mm. The experiment was performed in triplicate and distilled water was used as a negative control.

#### 2.2.2 Agar overlay method

Probiotic microorganisms (10 µL of 8 log CFU/mL) were dotted on MRS agar and incubated at 37°C overnight in anaerobic conditions. Afterward, the plate was poured with 5 mL of melted BHI agar containing *H. pylori* cells (8 log CFU/mL) and incubated overnight in anaerobic conditions. The inhibition zone was measured and reported as mm. The experiment was performed in triplicate.

### 2.3 Minimum inhibitory concentration, minimum bactericidal concentration, and fractional inhibitory concentration of postbiotics and derivative compounds

#### 2.3.1 Broth microdilution assay

As mentioned above, postbiotic solutions and derivative compounds (poly L-Lysine or glycerol monolaurate) were prepared. A compound of 100 µL (at a concentration of 1.2 mg/mL) was diluted by 2-fold dilution with 100 µL of BHI broth in a 96-well plate, and 100 µL of *H. pylori* cells (8 log CFU/mL) were added to the well. The plate was incubated overnight at 37°C in anaerobic conditions, and the last well to be inhibited (clear under vision) was recorded as the MIC value. All wells were dotted on BHI agar (Difco™, Sparks, MD, USA) to check *H. pylori* growth, and the last well showed no *H. pylori* growth, so it was recorded as the MBC value. As some strains did not exhibit a measurable MBC value, although inhibitory zones were observed, MBC values were determined using the following concentration series: 1.2, 1.1, 1.0, 0.9, 0.8, 0.7, and 0.6 mg/mL, respectively. The experiment was performed in triplicate.

#### 2.3.2 Checkerboard assay

Postbiotics derived from *L. paracasei* SD1 (postbiotic SD1), *L. rhamnosus* SD4 (postbiotic SD4), *L. rhamnosus* SD11 (postbiotic SD11), as well as poly L-Lysine and glycerol monolaurate, were tested for synergistic or antagonistic effects using a checkerboard assay. Each postbiotic solution, poly L-Lysine or glycerol monolaurate, was prepared at the concentrations of 1/12X, 1/6X, 1/4X, 1/2X, 1X, 2X, and 3X MIC, and 100 µL of each concentration was added into the 96-well plate. Then 100 µL of *H. pylori* (8 log CFU/mL) was added and the plate was incubated overnight at 37°C in anaerobic conditions. The lowest concentration of the combination of postbiotics, poly-L-lysine, or glycerol monolaurate showed growth inhibition compared to the untreated control. The accompanying equation calculated the fractional inhibitory concentration index (FIC index): FIC index = (MIC of solvent A in combination)/(MIC of solvent A alone) + (MIC of solvent B in combination)/(MIC of solvent B alone). The experiment was performed in triplicate.

### 2.4 Postbiotics, postbiotic-glycerol monolaurate, and poly L-Lysine-glycerol monolaurate mouthwash preparation

Mouthwashes were prepared in 3 formulations as follows: (i) postbiotic mouthwash containing 0.03 mg/mL postbiotic SD1 and 0.03 mg/mL postbiotic SD11, (ii) postbiotic-glycerol monolaurate mouthwash containing 0.02 mg/mL glycerol monolaurate along with 0.03 mg/mL postbiotic SD1 and 0.03 mg/mL postbiotic SD11, and (iii) poly L-lysine-glycerol monolaurate mouthwash containing 0.03 mg/mL poly L-lysine and 0.02 mg/mL glycerol monolaurate. All types of mouthwashes were used throughout the study and were freshly prepared before use. The mouthwash base comprising glycerol, kolliphor RH40, peppermint oil, saccharin sodium salt hydrate, methyl 4-hydroxybenzoate, and water (Wongsuwanlert et al., 2024b) was prepared and used as a negative control in all experiments.

### 2.5 Cell viability assay after being treated with postbiotics and postbiotic-glycerol monolaurate mouthwashes

H357, AGS, and PDL cells were used in this experiment. The cells were subcultured using the trypsinization method, and then the cells (3 x 10^4^ cells/mL) were seeded into 96-well plates. The culture plate was incubated for 72 h in a CO_2_ incubator and the cells showed approximately 99% cell confluence. An amount of 100 µL of postbiotic mouthwash or postbiotic-glycerol monolaurate mouthwash or poly L-Lysine-glycerol monolaurate mouthwash was added to monolayer cells for 5 min, 15 min, 1 h, 2 h, 6 h, and 24 h in a CO_2_ incubator. Cell viability was assessed using a MTT assay, and the percentage of cell viability was calculated by (OD 570 of the treated wells/OD 570 of non-treated wells) × 100.

### 2.6 Anti-adhesion ability of postbiotics and postbiotic-glycerol monolaurate mouthwashes

#### 2.6.1 Cell cultivation

Human oral squamous cell carcinoma (H357 was kindly derived from Professor Paul Speight of the University of Sheffield, UK) and human gastric adenocarcinoma cells (AGS, CRL-1739) were used in this study. H357 or AGS cells were cultured in cell culture flasks with Dulbecco’s modified Eagle’s medium (DMEM; Thermo Fisher Scientific, Waltham, MA, USA) supplemented with 10% fetal bovine serum (FBS; Thermo Fisher Scientific, Grand Island, NY, USA), 1% Penicillin-Streptomycin (Thermo Fisher Scientific, Grand Island, NY, USA), and 1% amphotericin B (Thermo Fisher Scientific, Grand Island, NY, USA) at 37°C in 5% CO_2_. The cells were subcultured using the trypsinization method, and then the cells (10^5^ cells/mL) were seeded into 24-well plates. The plate was incubated at 37°C in a CO_2_ incubator for 3 days, or until 95% cell confluence was reached, for the anti-adhesion study.

#### 2.6.2 Anti-adhesion assay

*H. pylori* cells (8 log CFU/mL) or a combination of *H. pylori* cells and postbiotic mouthwash or a combination of *H. pylori* cells and postbiotic-glycerol monolaurate mouthwash or a combination of *H. pylori* cells and poly L-Lysine-glycerol monolaurate mouthwash were added to the monolayer cells (Wongsuwanlert et al., 2024b). The plate was incubated at 37°C for 1 h and washed twice with PBS (pH 7.0). Trypsin-EDTA (0.25%) (Thermo Fisher Scientific, Canada) was added to remove adherent cells, and bacterial adhesion was counted by the plate count method. The percentage of adhesion ability was calculated using 100 x adherence bacteria/bacteria at the beginning. The experiment was conducted in triplicate, and the mouthwash base was used as the negative control.

### 2.7 Pro-inflammatory cytokines suppression after being treated with postbiotics and postbiotic-glycerol monolaurate mouthwashes

AGS and human periodontal ligament (PDL) cells were subcultured by the trypsinization method, and the cells (10^5^ cells/mL) were seeded in 6-well plates for 3 days or until 90% confluence. The cells were treated with an *H. pylori* strain and a combination of *H. pylori* and postbiotic mouthwash or *H. pylori* and postbiotic-glycerol monolaurate mouthwash or *H. pylori* and poly L-Lysine-glycerol monolaurate mouthwash for 24 h in a CO_2_ incubator. The cells were extracted for RNA using an RNA extraction kit (Thermo Fisher Scientific, CA, USA), and 2 µg RNA was synthesized for cDNA using a cDNA synthesis kit (Thermo Fisher Scientific, MA, USA). cDNA was measured for interleukin (IL)-1β, IL-6, IL-8, and TNF-α mRNA expression using the CFX96 TouchTM Real-Time PCR detection system (BioRad, Foster, CA, USA), and its conditions were followed according to Wongsuwanlert et al. (2024b). Glyceraldehyde 3-phosphate dehydrogenase was used as a housekeeping gene, and untreated cells were used as the control. The cytokine expression was normalized to the control (set to 1.0) and reported as a fold of induction. The experiment was performed in triplicate and the mouthwash base was used as a negative control.

### 2.8 Biofilm eradication of postbiotics and postbiotics-glycerol monolaurate mouthwashes using a microtiter biofilm formation assay

An *H. pylori* strain (8 log CFU/mL) of 200 µL was added to a 96-well plate and incubated at 37°C for 24 h in anaerobic conditions. The biofilm was washed twice with 200 µL PBS (pH 7.0) and exposed to 200 µL of postbiotics, postbiotic-glycerol monolaurate, and poly L-Lysine-glycerol monolaurate mouthwashes at 37°C for 5 min, 15 min, 1 h, 2 h, 6 h, and 24 h in anaerobic conditions. Biofilm eradication (%) was evaluated using a MTT assay and was confirmed using Live/Dead staining (Thermo Fisher Scientific, Eugene, OR, USA). The experiment was performed in triplicate, and the mouthwash base was used as a negative control.


$$\%\text{ biofilm eradication}=[1-\frac{\text{A}570\text{ of the treated wells}}{\text{A}570\text{ of the non}-\text{treated wells}}]\times 100$$

### 2.9 Determination of *cag*A suppression of postbiotics and postbiotics-glycerol monolaurate mouthwashes

Nineteen *H. pylori* strains (8 log CFU/mL) were incubated with a 50% lethal dose of postbiotic mouthwash or postbiotic-glycerol monolaurate mouthwash or poly L-Lysine-glycerol monolaurate mouthwash at 37°C for 24 h in anaerobic conditions. *H. pylori* cells were extracted for RNA using an RNA extraction kit (Thermo Fisher Scientific, CA, USA) following the instruction guidelines, and 100 ng/mL RNA was mixed with One Step real-time PCR solution (Meridian Bioscience, Memphis, TN, USA), nucleotide sequence primers (Macrogen, Korea), (Wongsuwanlert et al., 2024b) and distilled water. The reaction was used to determine *cag*A expression using the CFX96 TouchTM Real-Time PCR detection system (BioRad, Foster, CA, USA). The real-time PCR conditions were 40 cycles with a denaturing temperature of 95°C for 20s, annealing temperatures at 44°C for 20s, and a polymerizing temperature of 72°C for 25s. The *ure*A gene was used as a housekeeping gene in this study. *H. pylori* ATCC43504 was used as a control and the expression was set to 1.0. The experiment was performed in triplicate, and the mouthwash base was used as a negative control.

### 2.10 Stability test

Postbiotic mouthwash and postbiotic-glycerol monolaurate mouthwash were kept at 4°C and were tested for anti-*H. pylori*, biofilm eradication, and cell viability using the agar well diffusion method and MTT assay every 4 weeks for 24 weeks.

### 2.11 Statistical analysis

The results were expressed as mean ± SD for antibacterial activity, biofilm removal, cell viability, and pro-inflammatory cytokine suppression. A scatter plot with a mean value was displayed for the *cag*A mRNA expression and adhesion ability data. The differences between the groups were evaluated using the Mann-Whitney *U* test. A *p*-value < 0.05 indicated a significant difference in the data.

## 3 Results

### 3.1 Anti-*H. pylori* activity, MIC, MBC, and the FIC index of postbiotics and derivative compounds (poly L-Lysine and glycerol monolaurate)

An inhibition zone around the probiotic cells and postbiotics against *H. pylori* was observed, with the probiotic cells displaying a larger inhibition zone (28.61-31.21 mm) than postbiotics (15.21-20.00 mm, **Table 1**). Postbiotic SD11 (19.41 ± 0.83 mm for ATCC43504 and clinical strains) showed a greater zone than postbiotic SD1 (16.42 ± 0.40 mm for ATCC43504 and clinical strains) and postbiotic SD4 (15.61 ± 0.56 mm for ATCC43504 and clinical strains). Regarding the MIC value of postbiotics, the results showed that 0.20-0.33 mg/mL of postbiotic SD1, postbiotic SD4, and postbiotic SD11 inhibited the growth of *H. pylori* (ATCC43504 and clinical strains) while poly L-Lysine and glycerol monolaurate revealed 0.30 and 0.20 mg/mL, respectively. The results of *H. pylori* ATCC43504 showed alignment with the oral clinical strains. A synergistic effect (FIC index ≤ 0.5) was observed with postbiotic SD1 and postbiotic SD11, as well as between postbiotic SD1 and glycerol monolaurate and between postbiotic SD11 and glycerol monolaurate (**Table 2**). Therefore, two types of mouthwashes were used throughout the study: one containing postbiotic SD1 and postbiotic SD11 (postbiotic mouthwash) and another combining postbiotic SD1, postbiotic SD11, and glycerol monolaurate (postbiotic-glycerol monolaurate mouthwash). Additionally, poly L-lysine-glycerol monolaurate mouthwash was used for comparison.

**Table 1:** Mean ± SD of antimicrobial activity of probiotics and postbiotics against *Helicobacter pylori* ATCC43504 and 19 strains of oral *H. pylori*.

Probiotics/Postbiotics/ Derivative compounds	Inhibition zone (mm)		MIC (mg/mL)		MBC (mg/mL)	
	ATCC43504	19 clinical strains	ATCC43504	19 clinical strains	ATCC43504	19 clinical strains
Probiotics						
SD1	30.00 ± 0.00	29.92 ± 1.21^b^	N/A	N/A	N/A	N/A
SD4	29.03 ± 0.01	28.61 ± 1.81^b^	N/A	N/A	N/A	N/A
SD11	31.00 ± 0.00	31.21 ± 2.02^a^	N/A	N/A	N/A	N/A
Postbiotics						
SD1	16.70 ± 0.10	16.14 ± 1.41^b^	0.20 ± 0.00	0.33 ± 0.09^a^	0.80 ± 0.00	0.85 ± 0.11^b^
SD4	16.00 ± 0.30	15.21 ± 2.52^b^	0.20 ± 0.00	0.33 ± 0.09^a^	1.00 ± 0.00	1.10 ± 0.21^a^
SD11	20.00 ± 0.20	18.82 ± 2.92^a^	0.20 ± 0.00	0.33 ± 0.09^a^	0.70 ± 0.00	0.75 ± 0.21^b^
Derivative compounds						
Glycerol monolaurate	30.20 ± 0.60	30.51 ± 0.32^a^	0.20 ± 0.00	0.20 ± 0.00^a^	0.20 ± 0.00	0.20 ± 0.00^a^
Poly L-Lysine	25.30 ± 0.40	26.50 ± 0.22^b^	0.30 ± 0.00	0.30 ± 0.00^a^	0.30 ± 0.00	0.30 ± 0.00^a^

N/A, not applicable; Lowercase letters showed statistical differences in antimicrobial activity of oral clinical strains, MIC, and MBC values between probiotic strains or derivative compounds (*p* < 0.05).

**Table 2:** Mean ± SD of the synergistic effect of postbiotics and derivative compounds against *Helicobacter pylori* strains.

Combinations	Mean ± SD of FIC index (20 *H. pylori* strains)
SD1 and SD4	0.94 ± 0.31
SD1 and SD11	0.22 ± 0.03*
SD4 and SD11	0.74 ± 0.04
SD1 and Glycerol monolaurate	0.22 ± 0.03*
SD4 and Glycerol monolaurate	1.33 ± 0.01
SD11 and Glycerol monolaurate	0.22 ± 0.03*
SD1 and Poly L-lysine	4.00 ± 0.03
SD4 and Poly L-lysine	4.00 ± 0.04
SD11 and Poly L-lysine	3.02 ± 0.04
Glycerol monolaurate and Poly L-lysine	0.20 ± 0.00*

FIC index ≤ 0.5 synergy; FIC index > 0.5 to 4 indifferences; FIC index > 4 antagonism.

Asterisk showed a synergistic effect.

### 3.2 Epithelial cell viability after exposure to mouthwashes

Epithelial cell viability depended on the incubation time, with 5 min (79.78%-91.56% for H357, 100.00%-100.00% for PDL, and 77.23%-80.21% for AGS) and 15 min (53.89%-83.93% for H357, 100.00%-100.00% for PDL, and 61.20%-75.23% for AGS) showing the highest cell viability. PDL cells showed 100.00% cell viability until 15 min compared to other cells, followed by H357 and AGS cells (**Table 3**). Postbiotic-glycerol monolaurate mouthwash showed the highest cell viability compared to the postbiotic- and poly L-Lysine-glycerol monolaurate mouthwashes at all times and cells tested.

**Table 3:** Mean ± SD of cell viability of human oral epithelial cells (H357), human periodontal ligament cells (PDL), and human gastric cancer cells (AGS) after being treated with postbiotic-, postbiotic-glycerol monolaurate-, and poly L-Lysine-glycerol monolaurate- mouthwashes.

Mouthwash formulations	Cell survival rate (%)					
	5min	15 min	1 h	2 h	6 h	24 h
H357 cells						
Mouthwash base	67.04 ± 1.07^a,D^	47.78 ± 0.34^b,D^	27.04 ± 1.97^c,D^	16.21 ± 0.78^d,D^	12.04 ± 0.51^e,D^	10.44 ± 0.68^f,D^
Postbiotic-glycerol monolaurate	91.56 ± 2.86^a,A^	83.93 ± 0.60^b,A^	71.97 ± 2.01^c,A^	60.76 ± 1.03^d,A^	56.90 ± 0.81^e,A^	42.54 ± 0.80^f,A^
Postbiotics	83.39 ± 1.32^a,B^	78.81 ± 0.03^b,B^	67.69 ± 0.43^c,B^	55.96 ± 0.42^d,B^	52.61 ± 0.23^e,B^	38.22 ± 0.22^f,B^
Poly L-Lysine-glycerol monolaurate	79.78 ± 2.91^a,C^	53.89 ± 0.60^b,C^	32.04 ± 2.02^c,C^	20.82 ± 1.01^d,C^	16.91 ± 0.83^e,C^	12.49 ± 0.81^f,C^
PDL cells						
Mouthwash base	79.30 ± 0.71^a,B^	74.84 ± 0.21^b,B^	63.53 ± 0.33^c,C^	52.24 ± 0.61^d,D^	41.44 ± 0.69^e,D^	22.14 ± 0.91^f,D^
Postbiotic-glycerol monolaurate	100.00 ± 1.40^a,A^	100.00 ± 1.51^a,A^	98.43 ± 2.22^b,A^	92.84 ± 1.60^c,A^	75.57 ± 1.55^d,A^	45.63 ± 1.20^e,A^
Postbiotics	100.00 ± 0.81^a,A^	100.00 ± 0.91^a,A^	93.10 ± 0.56^b,C^	80.51 ± 0.04^c,C^	53.26 ± 0.04^d,C^	33.32 ± 0.62^e,C^
Poly L-Lysine-glycerol monolaurate	100.00 ± 0.03^a,A^	100.00 ± 0.04^a,A^	95.40 ± 0.02^b,B^	82.81 ± 0.04 ^c,B^	55.59 ± 0.01^d,B^	35.64 ± 0.04 ^e,B^
AGS cells						
Mouthwash base	63.02 ± 0.60^a,C^	48.44 ± 0.40^b,D^	33.93 ± 3.21^c,D^	24.00 ± 0.39^d,D^	16.40 ± 0.41^e,D^	4.33 ± 0.61^f,D^
Postbiotic-glycerol monolaurate	80.21 ± 1.10^a,A^	75.23 ± 2.56^b,A^	62.33 ± 1.91^c,A^	54.54 ± 2.54^d,A^	40.45 ± 1.60^e,A^	27.41 ± 0.63^f,A^
Postbiotics	77.89 ± 0.45^a,B^	68.91 ± 1.02^b,B^	48.04 ± 0.31^c,B^	42.21 ± 0.90^d,B^	31.21 ± 0.03^e,B^	19.10 ± 0.04^f,B^
Poly L-Lysine-glycerol monolaurate	77.23 ± 1.41^a,B^	61.20 ± 1.51^b,C^	42.32 ± 1.11^c,C^	34.51 ± 1.60^d,C^	28.54 ± 1.61^e,C^	17.43 ± 2.11^f,C^

Capital letters showed statistical differences between groups at the same time; lowercase letters showed statistical differences between times in the same group (*p* < 0.05).

### 3.3 Anti-adhesion effects by mouthwashes

**Figure 1:**
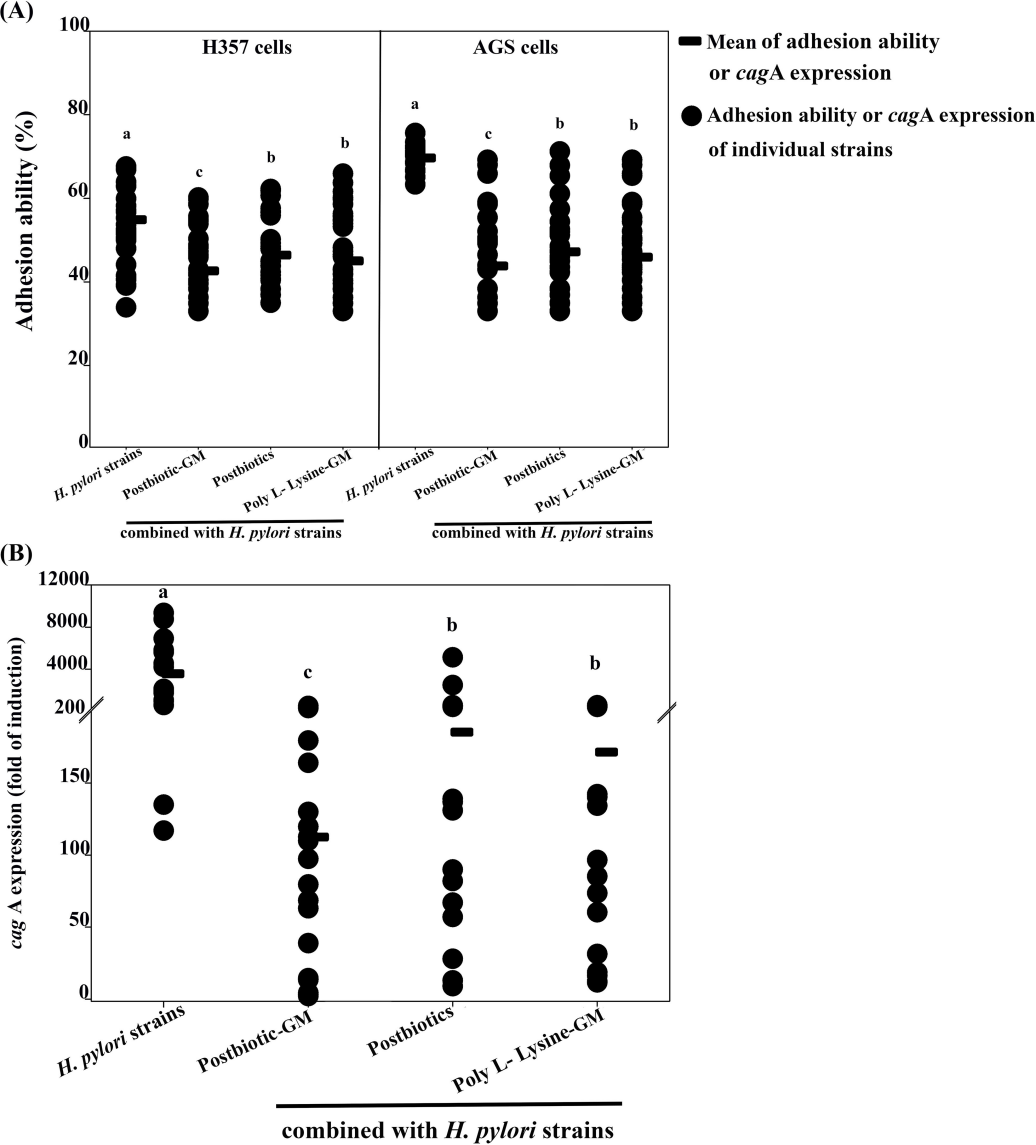
Graph **(A)** shows the adhesion ability of H357 and AGS cells to *Helicobacter pylori* strains and their combinations with postbiotic-glycerol monolaurate (Postbiotic-GM), postbiotics, and poly L-Lysine-glycerol monolaurate (Poly L-Lysine-GM). Each group shows the mean adhesion and individual data points. Graph **(B)** depicts *cagA* expression (fold of induction) with the same combinations, showing mean expression and individual data points. Each category has letters a, b, or c indicating significant differences.

No cell cytotoxicity was found after testing with either *H. pylori* or the mouthwashes. A decrease in the adhesion of *H. pylori* strains was observed on H357 and AGS cells, particularly with the postbiotic-glycerol monolaurate mouthwash, which demonstrated the highest adhesion reduction (12.25% for H357 cells and 25.82% for AGS cells; **Figure 1A**). The postbiotic mouthwash resulted in an adhesion reduction of 8.47% for H357 cells and 22.44% for AGS cells, while the poly L-Lysine-glycerol monolaurate mouthwash showed a decrease of 9.88% for H357 cells and 23.72% for AGS cells. Both mouthwashes showed no significant differences, but both resulted in adhesion reductions that were significantly lower than that of *H. pylori* alone (*p* < 0.05).

### 3.4 Pro-inflammatory cytokines suppression by mouthwashes

Neither *H. pylori* nor the mouthwashes showed cytotoxicity against the cells tested in the experiment. The inflammation results indicated that AGS cells expressed more pro-inflammatory cytokines than PDL cells. All mouthwashes showed low levels of pro-inflammation stimulation in PDL (IL-1β at 0.53-0.91 fold, IL-6 at 0.61-1.02 fold, IL-8 at 3.42-5.71 fold, and TNF-α at 0.43-0.63 fold) and AGS cells (IL-1β at 1.37-1.62 fold, IL-6 at 1.87-2.27 fold, IL-8 at 7.42-8.51 fold, and TNF-α at 1.31-1.45 fold), whereas *H. pylori* strains (*H. pylori* ATCC43504 and 19 clinical strains) stimulated both cells to express high levels of pro-inflammatory cytokines (IL-1β at 2.04-3.24 fold, IL-6 at 3.08-4.63 fold, IL-8 at 20.23-46.51 fold, and TNF-α at 3.54-5.45 fold). Among the pro-inflammatory cytokines, IL-8 showed the highest cytokine expression, followed by TNF-α, IL-6, and IL-1β. However, pro-inflammatory cytokines were reduced after being combined with the mouthwashes. The postbiotic mouthwash revealed a high reduction of pro-inflammatory cytokines compared to others (**Table 4**), and IL-8 showed the highest significant decrease in both cell types (3.03-5.21 fold for PDL cells and 16.79-28.51 fold for AGS cells, *p* < 0.05).

**Table 4:** Mean ± SD of pro-inflammatory cytokine stimulation by *Helicobacter pylori* (ATCC43504 and 19 strains of oral *H. pylori*) or by each *H. pylori* strain and mouthwash on AGS and PDL cells.

Mouthwashes	Pro-inflammatory cytokines (folds of induction)				
	IL-1β	IL-6	IL-8	TNF-α
AGS cells				
Mouthwash base	3.11 ± 0.45^d,A^	18.09 ± 0.40^c,A^	257.03 ± 0.30^a,A^	73.78 ± 6.91^b,A^
*H. pylori* strains	3.24 ± 0.12^d,A^	4.63 ± 0.20^c,B^	46.51 ± 8.89^a,B^	5.45 ± 0.70^b,B^
Postbiotic-glycerol monolaurate and *H. pylori*	2.02 ± 0.21^c,B^	2.67 ± 0.13^b,C^	18.32 ± 0.31^a,D^	2.53 ± 0.22^b,C^
Postbiotics and *H. pylori*	1.67 ± 0.41^c,C^	1.13 ± 0.04^d,D^	18.00 ± 1.00^a,D^	2.31 ± 0.04^b,C^
Poly L-Lysine-glycerol monolaurate and *H. pylori*	1.73 ± 0.60^c,C^	3.54 ± 1.44^b,C^	29.72 ± 9.30^a,C^	3.52 ± 1.40^b,C^
PDL cells					
Mouthwash base	2.72 ± 0.21^b,A^	2.78 ± 0.22^b,A^	22.82 ± 0.22^a,A^	2.73 ± 0.21^b,B^
*H. pylori* strains	2.04 ± 0.20^c,B^	3.08 ± 0.31^b,A^	20.23 ± 1.41^a,B^	3.54 ± 0.31^b,A^
Postbiotic-glycerol monolaurate and *H. pylori*	1.44 ± 0.41^b,C^	2.02 ± 0.72^b,C^	15.89 ± 1.01^a,C^	1.89 ± 0.78^b,D^
Postbiotics and *H. pylori*	1.40 ± 0.67^c,C^	1.84 ± 1.00^b,C^	15.02 ± 2.14^a,C^	2.09 ± 1.41^c,D^
Poly L-Lysine-glycerol monolaurate and *H. pylori*	1.70 ± 0.67^c,C^	2.40 ± 0.41^b,B^	17.20 ± 5.67^a,D^	2.42 ± 0.62^c,C^

Capital letters showed statistical differences between groups at the same time; lowercase letters showed statistical differences between times in the same group (*p* < 0.05).

### 3.5 Biofilm eradication by mouthwashes

**Figure 2:**
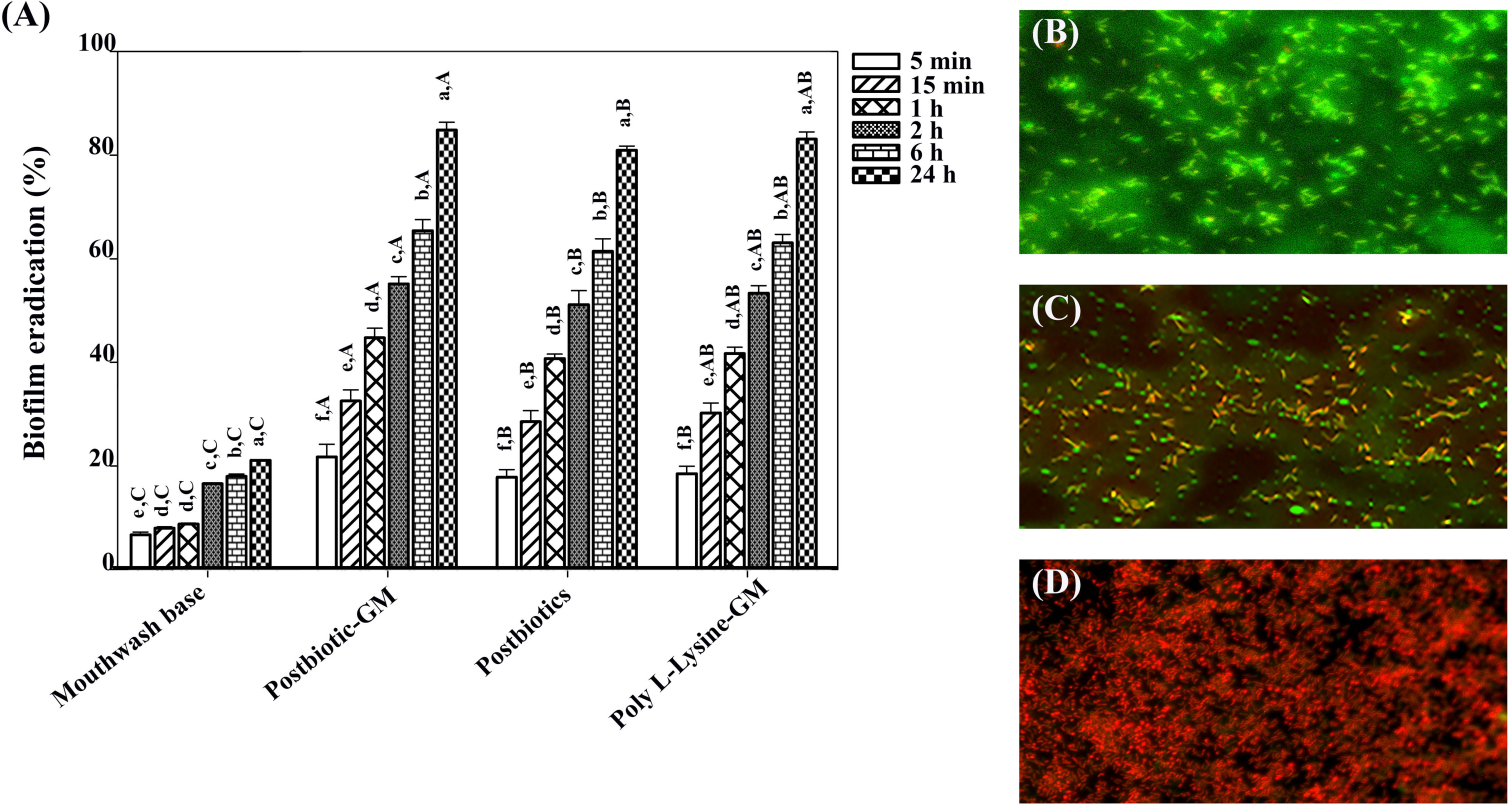
**(A)** Bar chart showing biofilm eradiation percentages for different treatments over various time intervals. Capital letters indicate significant differences between groups at the same time point, while lowercase letters indicate significant differences between time points within the same group (*p* < 0.05). **(B)** Micrograph with green fluorescence indicating biofilm presence. **(C)** Micrograph showing red and green fluorescence demonstrating biofilm disruption. **(D)** Micrograph with dominant red fluorescence, suggesting extensive biofilm eradication.

**Figure 2A** demonstrated that the biofilm was effectively removed after treatment with all mouthwashes. Importantly, postbiotic-glycerol monolaurate mouthwash showed a higher efficacy (21.30%-84.37%) in biofilm removal compared to postbiotic mouthwash (17.43%-80.51%) and poly L-Lysine-glycerol monolaurate mouthwash (18.03%-82.59%). Biofilm eradication was time-dependent, with the most effective eradication observed after 24 h of incubation with all mouthwashes (84.44%, 80.52%, and 82.57% for postbiotics-glycerol monolaurate mouthwash, postbiotic mouthwash, and poly L-Lysine-glycerol monolaurate mouthwash, respectively). Biofilm removal was confirmed with live/dead staining (**Figures 2B–D**). After treating the biofilm with postbiotics-glycerol monolaurate mouthwash for 24 h, a significant proportion of the biofilm was found dead (red color) compared to the untreated cells (green color).

### 3.6 *cag*A suppression by mouthwashes

The study results indicate that *H. pylori* treated with the mouthwashes demonstrated a notable decrease in *cag*A expression compared to the strain without treatment (**Figure 1B**). Among the mouthwashes tested, the postbiotic-glycerol monolaurate mouthwash resulted in the highest reduction of *cag*A expression, achieving a remarkable 3467.45-fold decrease. This was closely followed by the poly L-Lysine-glycerol monolaurate mouthwash, which resulted in a 3408.50-fold reduction, and the postbiotic mouthwash, which showed a 3394.61-fold reduction in *cag*A expression.

### 3.7 Stability of postbiotic mouthwash and postbiotic-glycerol monolaurate mouthwash

The effects of both types of mouthwashes after 24 weeks showed a decrease compared to baseline levels. At the baseline, the anti-*H. pylori* activity, biofilm eradication, and epithelial cell viability for the postbiotic mouthwash were 20.04 mm, 78.03%, and 83.33%, respectively, while the postbiotic-glycerol monolaurate mouthwash had the corresponding values of 30.01 mm, 80.03%, and 91.58%, respectively. However, after 24 weeks, these parameters declined to 14.67 mm and 16.31 mm for anti-*H. pylori* activity, with biofilm eradication and cell viability dropping to 32.11% and 34.92%, as well as 35.92% and 39.45%, respectively, for both mouthwashes.

## 4 Discussion

*Helicobacter pylori* infection in the stomach is usually treated with a combination of drugs (clarithromycin and either amoxicillin or metronidazole) along with bismuth (Chey and Wong, 2007). The oral cavity may serve as a potential reservoir for *H. pylori*, allowing transmission to other sites in the human body and leading to the recurrence of *H. pylori* gastritis (Wongsuwanlert et al., 2024). Therefore, a comprehensive treatment aimed at eliminating *H. pylori* in both the stomach and oral cavity may help more effectively eradicate *H. pylori* gastric infection. The study found that postbiotic mouthwash and postbiotic-glycerol monolaurate mouthwash reduced virulence factors of *H. pylori* including its adhesion to host cells, pro-inflammatory cytokine production, biofilm formation, and *cag*A expression, while also demonstrating lower toxicity to host cells. The reduction effects of mouthwashes against clinical oral *H. pylori* were found for up to 24 weeks of storage.

After mouthwash formulation, the mouthwashes were assessed for toxicity on host cells, showing a time-dependent toxic effect. At 24 h of exposure, all tested cell types presented more than 50% toxicity, which based on the ISO 10993-5:2009 standard, can be classified as highly cytotoxic. However, typical daily mouthwash use does not exceed 5 min, indicating that the observed cytotoxicity at 24 h may not accurately reflect the *in vivo* exposure conditions during regular oral use. Our study revealed that postbiotic-glycerol monolaurate exhibited low cytotoxicity, with 0-17% cytotoxicity observed in oral cells (keratinocyte and periodontal ligament cells) and 25% in gastric epithelial cells after 15 min of incubation. According to the standard, reports cell cytotoxicity as < 30%, is classified as slightly cytotoxic. This level is generally considered acceptable for biomaterial applications, particularly when the exposure is short-term or in direct or prolonged contact with sensitive tissues. Conversely, mouthwash base displayed high cell cytotoxicity (26-53%), despite the mouthwash base adding glycerol to maintain hydration, enhancing barrier integrity, and reducing cytotoxic responses (Kvalheim et al., 2019). Postbiotics can inhibit the death of gut epithelial cells and promote their growth. They assist in tissue repair and enhance goblet cell development, which produces mucus to protect intestinal epithelial cells and maintain their integrity. A previous study showed that postbiotics exhibited low toxicity even after 15 days of use, with no significant changes in liver enzyme levels or intestinal histopathology in mice (Pesarico et al., 2023). Studies revealed that both glycerol monolaurate (at concentrations less than 640 µM) and postbiotics (at concentrations below 1000 µg/mL) exhibited no cytotoxicity toward critical immune cells including dendritic cells, human gingival fibroblast cells, and human peripheral blood mononuclear cells (Poulsen et al., 2015; Silva et al., 2018; Pahumunto et al., 2020). Notably, glycerol monolaurate has been shown to stabilize eukaryotic cell membranes, neutralize bacterial exotoxin toxicity, and affect anti-bactericidal activity. With such evidence, integrating postbiotic-glycerol monolaurate mouthwash into oral care routines is a promising approach for promoting oral health (Pesarico et al., 2023).

Postbiotics-glycerol monolaurate mouthwash demonstrated strong anti-adhesion activity on oral keratinocyte cells (H357) and gastric epithelial cells (AGS). Our study found that the mouthwash inhibited the adhesion of *H. pylori* strains to AGS cells more effectively than to H357 cells, which was likely due to *H. pylori* stronger tendency to adhere to gastric epithelial cells compared to H357 cells. The anti-adhesion effect is likely attributed to the ability of postbiotics or probiotics to inhibit the initial colonization of pathogens by enhancing antimicrobial peptides in the body and forming protective layers that prevent pathogen adhesion and invasion (Song et al., 2019). Previous studies reported that live lactobacilli, *L. acidophilus*, *L. bulgaricus, L. paracasei* SD1*, L. rhamnosus* SD4*, and L. rhamnosus* SD11, and dead lactobacilli could inhibit *H. pylori* adherence to gastric epithelial cell line-1, AGS cells, and human gastric adenocarcinoma cell lines (Che et al., 2024; Xu et al., 2022; Song et al., 2019). For instance, certain *Lactobacillus* strains can bind to toll-like receptors on host cells, thereby inhibiting the adhesion of pathogenic bacteria to gastric mucosal epithelial cells (Song et al., 2019). Specifically, *L. reuteri* secretes adhesion factors that competitively block *H. pylori* adhesion by binding to the receptor sites on gastric mucosal epithelial cells (Song et al., 2019). The evidence clearly shows that probiotics and postbiotics are crucial in preventing pathogens from adhering and invading host cells. Nonetheless, the mechanism by which the mouthwash inhibits *H. pylori* adhesion remains unclear and requires further clarification.

Virulence genes of *H. pylori*, especially *cag*A and *vac* genes, are associated with the severity of gastritis or gastric cancer. The most studied virulence-associated component of *H. pylori* is the *cag*A protein, which enters host gastric epithelial cells through the type 4 secretion system (Azizimoghaddam et al., 2023). Our study observed variability in *cag*A expression among *H. pylori* strains, potentially linked to differences in pathological outcomes; however, the underlying mechanisms remain unclear. This suggests that strain-specific factors may contribute to the upregulation of *cag*A expression. A previous study showed that a mouthwash combining poly L-Lysine and glycerol monolaurate successfully reduced *cag*A gene expression in *H. pylori* strains (Wongsuwanlert et al., 2024b). However, the impact of postbiotics on *cag*A suppression remains unexplored. Our study demonstrates that postbiotics and postbiotic-glycerol monolaurate mouthwashes significantly inhibit *cag*A expression in *H. pylori* strains following co-incubation. Additionally, lactobacilli cell-free supernatants effectively diminished the expression of the primary exotoxins produced by *Aggregatibacter actinomycetemcomitans* (leukotoxin and CDT) and downregulated *kat*A, thereby decreasing the bacterium’s survival in the presence of H_2_O_2_ (Ishikawa et al., 2021). This compelling evidence highlights the potential of postbiotics in combating harmful bacterial strains. The reduction in *cag*A expression was associated with a decrease in pro-inflammatory cytokines, particularly IL-8. Our study also found that postbiotics and postbiotic-glycerol monolaurate mouthwashes can downregulate pro-inflammatory cytokines in oral and gastric cells after stimulation with *H. pylori* strains. This finding aligns with a previous study showing that the cell-free supernatant of these strains can suppress IL-8 stimulation in oral and intestinal cells exposed to *Porphyromonas gingivalis* (Pahumunto and Teanpaisan, 2023). This suppression may be due to the crucial role of postbiotics in inhibiting the phosphorylation and translocation of *cag*A (Ma et al., 2023; Lin et al., 2009). Additionally, glycerol monolaurate can modulate the production of pro-inflammatory cytokines in eukaryotic cells (Wongsuwanlert et al., 2024b).

Growth inhibition and biofilm eradication are recommended to eliminate or reduce *H. pylori* in the body; these functions are important to protect against infection by this organism. Previous studies have shown that poly-L-lysine and glycerol monolaurate, in mouthwash form, inhibited the growth of *H. pylori* and decreased the number of *H. pylori* in the oral cavity of volunteers (Wongsuwanlert et al., 2024a; Wang et al., 2014). However, controlling the quality of these compounds can be challenging. This study examined postbiotics as alternatives to chemical compounds and found that postbiotic mouthwash and postbiotic-glycerol monolaurate mouthwash showed the highest capacity to eliminate *H. pylori* in both planktonic and biofilm forms. Surprisingly, postbiotics exhibited close inhibitory effects against both *H. pylori* ATCC 43504 and clinical strains, possibly due to their shared mechanisms of action in eradicating *H. pylori*. The mechanisms and capacities of postbiotics, specifically cell-free supernatants, containing various bioactive compounds such as antimicrobial proteins (Wannun et al., 2014), antioxidants (Chooruk et al., 2017), and short-chain fatty acids, specifically acetic acid, propionic acid, and butyric acid, have been identified (Thananimit et al., 2022). These substances can destroy bacterial cell walls, lead to bacterial death, and interfere with bacterial adhesion to host cells (Song et al., 2019). While research on postbiotics is still emerging, a few studies have specifically investigated their impact on *H. pylori* infection (Ma et al., 2023; Yang et al., 2024). In contrast, numerous studies have focused on the effects of probiotics on *H. pylori* strains. These studies have shown that probiotics such as *Lactobacillus helveticus*, *L. acidophilus*, *L. brevis*, *L. casei*, *L. johnsonii*, *L. rhamnosus*, *Bifidobacterium*, *Streptococcus thermophilus*, *S. faecalis*, *Lactococcus*, and *S. intermedia* can inhibit *H. pylori* growth, alleviate clinical symptoms, enhance the effectiveness of *H. pylori* eradication therapy, and reduce adverse drug reactions (McFarland et al., 2016; Yang et al., 2024). Although these studies primarily examine probiotics, recent reports suggest that postbiotics may have similar effects against pathogens (*Porphyromonas gingivalis*, *Fusobacterium nucleatum*, *Salmonella enterica*), including *H. pylori* (Juntarachot et al., 2023; Pahumunto and Teanpaisan, 2023).

A stability test of the mouthwashes was modified to allow storage at 4°C for 6 months, which is applicable to aqueous-based pharmaceutical formulations. After 24 weeks of storage, postbiotic-glycerol monolaurate mouthwashes demonstrated a notable decline in biological activities (anti-*H. pylori*, anti-biofilm, and cell viability) compared to their baselines. The reduction of biological activities may be the result of a loss of active antimicrobial metabolites, likely due to degradation over time, and the functional integrity of key postbiotic components, such as short-chain fatty acids and anti-microbial proteins, may have declined. These results emphasize the importance of optimizing formulation stability to preserve biological efficacy over extended storage periods.

In conclusion, the present study found that postbiotics-glycerol monolaurate mouthwash effectively reduced *H. pylori* adhesion to host cells and diminished pro-inflammatory cytokine stimulation. This mouthwash also demonstrated the ability to remove biofilm and regulate *cag*A expression. Mouthwash containing postbiotics shows promise as a preventative strategy against *H. pylori* infection and may serve as adjuvant therapy for gastrointestinal disorders linked to *H. pylori* infection, considering their low toxicity and health benefits. Further research is needed to investigate the impact of postbiotic-glycerol monolaurate mouthwash on reducing *H. pylori* and its virulence factors in a clinical study. In addition, the formulation should be further optimized to improve its stability during long-term storage, ensuring the preservation of its beneficial properties and therapeutic efficacy.

## Data Availability

The raw data supporting the conclusions of this article will be made available by the authors, without undue reservation.
